# Supporting people in mental health crisis in 21st-century Britain

**DOI:** 10.1192/bjb.2019.93

**Published:** 2020-12

**Authors:** Andrew Molodynski, Stephen Puntis, Em Mcallister, Hannah Wheeler, Keith Cooper

**Affiliations:** 1 Oxford Health NHS Foundation Trust, UK; 2 Oxford University, UK; 3 Lived Experience Advisor, UK; 4 Her Majesty's Inspectorate of Constabulary and Fire and Rescue Services, UK; 5 The Doctor Magazine, British Medical Association, UK

**Keywords:** Stigma and discrimination, crisis care, mental health services, policing and mental health, mental health funding

## Abstract

Recent years have seen a surge in interest in mental healthcare and some reduction in stigma. Partly as a result of this, alongside a growing population and higher levels of societal distress, many more people are presenting with mental health needs, often in crisis. Systems that date back to the beginning of the National Health Service still form the basis for much care, and the current system is complex, hard to navigate and often fails people. Law enforcement services are increasingly being drawn into providing mental healthcare in the community, which most believe is inappropriate. We propose that it is now time for a fundamental root and branch review of mental health emergency care, taking into account the views of patients and the international evidence base, to ‘reset’ the balance and commission services that are humane and responsive – services that are fit for the 21st century.

Recent years have seen a surge of interest in mental healthcare. Media columns, royal endorsements, prominence in policy and legislative agenda all point to the gradual erosion of stigma surrounding mental illness. However, the most recent Care Quality Commission report on mental health services showed that services still struggle to provide people with the help they need, with 40% of National Health Service (NHS) providers being rated as poor or requiring improvement in the most fundamental domain of all, safety.^1^ Although this is widely acknowledged, it has not as yet been remedied and is particularly problematic in the management of so-called mental health crises.

Emergency medical services still operate on the historically established principles developed during the birth of the NHS, with a single means of emergency transport to large accident and emergency (A&E) departments staffed and equipped to largely deal with physical problems. This was entirely understandable in an era when few treatments were available for mental health crises and few people presented to services, but it has become increasingly outdated over time. A significant and rising proportion of A&E attenders come with a mental health crisis of some sort.^2,3^ Many community mental health services (for all age groups) are increasingly stretched owing to a period of significantly rising demand, and the relatively flat workforce is leading to more people presenting in crisis as we are less able to intervene early and avoid such presentations. It is important to bear this wider picture in mind while thinking about emergency mental healthcare.

There have been welcome developments in emergency mental healthcare, including crisis teams,^4,5^ but these are generally restricted to those already under secondary mental healthcare, with only one service in England to our knowledge being genuinely ‘open access’, with people able to just turn up and be seen. Otherwise, people must go through a general practitioner, the police, or A&E. Indeed, many crisis services have shrunk in recent years, with providers being ‘freed’ from adhering to the evidence-based care of the national service framework and operating under reduced funding.^6^ Many struggle to offer a fast and flexible service to those in need in the face of increasing demand, and many people are discharged early from hospital or not admitted owing to chronic NHS mental health bed shortages.

Other developments have included police street triage services,^7^ which despite their name often support people in mental health crises in their own homes as well as in public places. Their introduction has come alongside a welcome drop in the use of police custody for up to 24 h under section 136 of the Mental Health Act, although overall section 136 use has not seen such reductions.^8^ However, these welcome reductions in police custody use are not universal and cannot solely be attributed to triage services, as both legislation and policy have also substantially changed.^9^ Street triage services vary markedly; while being part of a crucial safety net, they operate alongside police officers, which consumes significant police time and means that for many the response to their health crisis comes in a police car – a marked contrast to physical care, and distressing and stigmatising for many.

Other welcome initiatives include places where people in crisis can drop in and receive support, generally from staff employed in the charity/third sector. Terminology varies (crisis café, safe haven, etc.), as do mode and hours of operation. Again, provision is variable and few such places, if any, are open 24/7. We could find no empirical evidence for their effectiveness or specific service models or components of models.

The result of all the above is a patchwork of provision developed during isolated attempts to patch up the problem of mental health crisis response rather than developing system-wide solutions. They make crisis care hard to negotiate for anyone, let alone people in distress and their families. It is widely agreed that the current evidence base is poor.^2,10–12^ We need to ask ourselves some fundamental and difficult questions. Are busy A&E departments the right place for those in crisis, such as those at risk of suicide or self-harm, or those with symptoms of psychosis? Are uniformed police officers the correct response to people in distress who have not acted unlawfully? Why are there so many cracks in provision that people can, and do, fall through during crisis?

We believe a root and branch review of crisis services based upon evidence from this country and overseas is needed. This should be guided by those who receive services and their families, alongside those who provide them. Such a process could move our mental health crisis services (belatedly) into the 21st century. We need to be bold. Initial evaluations of dedicated mental health ambulances in Sweden that work on a similar basis to their physical counterparts are encouraging, with much demand and favourable reports of patient experience.^13^ In Australia, projects of peer-to-peer support with care packages to avoid hospital admission have shown encouraging results.^14^ We need to evaluate the current movement of crisis cafes and safe havens rigorously, both in terms of effectiveness and of models that work (or don't).

The NHS long-term plan (www.longtermplan.nhs.uk) has an ambitious vision for emergency mental health services in particular, with improved access and greater patient focus. It also promises a bolstering of services for people with severe and enduring mental illnesses, and that out of area placements (OAPs) will end. Unfortunately, a very recent NHS report^15^ has shown that OAPs are actually rising again. However, detailed analysis of spending commitments and the current worsening workforce problems in all disciplines makes it clear that these worthy ambitions cannot be realised without a step change in our thinking about and our valuing of mental healthcare. This, alongside the review of mental health legislation and the universal acknowledgment that services are inadequate, means that now is the time to invest in the future by taking a considered and evidence-based approach to decide what should be available to provide the best experiences of care and the best outcomes. To continue with our ongoing *ad hoc*, piecemeal approach does our patients a disservice.

## About the authors

**Andrew Molodynski** is the mental health policy lead for the British Medical Association Consultant Committee and a full-time NHS clinical psychiatrist, Oxford Health NHS Foundation Trust and Oxford University, Oxford, UK. **Stephen Puntis** is a senior researcher with an interest in mental health service provision in the Department of Psychiatry, Oxford University, Oxford, UK. **Em Mcallister** is a Lived Experience Advisor, UK. **Hannah Wheeler** is a Metropolitan Police Service officer seconded to Her Majesty's Inspectorate of Constabulary and Fire and Rescue Services, London, UK. **Keith Cooper** is a senior staff writer at The Doctor Magazine, British Medical Association, London, UK.

## References

[1] Care Quality Commission. The State of Care in Mental Health Services 2014 to 2017. Findings from CQC's Programme of Comprehensive Inspections of Specialist Mental Health Services. CQC, 2018.

[2] Barratt H, Rojas-García A, Clarke K, Moore A, Whittington C, Stockton S, Epidemiology of mental health attendances at emergency departments: systematic review and meta-analysis. PLoS ONE 2016; 11(4): e0154449. 27120350 10.1371/journal.pone.0154449 PMC4847792

[3] Lowthian JA, Curtis AJ, Cameron PA, Stoelwinder JU, Cooke MW, McNeil JJ. Systematic review of trends in emergency department attendances: an Australian perspective. Emerg Med J 2011; 28(5): 373–7. 20961936 10.1136/emj.2010.099226

[4] Department of Health. National Service Framework for Mental Health. Modern Standards and Service Models. Department of Health, 1999 (https://www.gov.uk/government/publications/quality-standards-for-mental-health-services).

[5] Glover G, Arts G, Babu KS. Crisis resolution/home treatment teams and psychiatric admission rates in England. Br J Psychiatry 2006; 189(5): 441–5. 17077435 10.1192/bjp.bp.105.020362

[6] Gilburt, H. Mental Health Under Pressure. The King's Fund, 2015 (https://www.kingsfund.org.uk/sites/default/files/field/field_publication_file/mental-health-under-pressure-nov15_0.pdf).

[7] Kirubarajan A, Puntis S, Perfect D, Tarbit M, Buckman M, Molodynski A. Street triage services in England: service models, national provision and the opinions of police. BJPsych Bull 2018; 42(6): 253–7. 30221614 10.1192/bjb.2018.62 PMC6465222

[8] National Police Chiefs' Council Lead for Mental Health. *Use of Section 136 of the Mental Health Act 1983 in 2015–16 (England and Wales)* NPCC, 2016 (https://www.npcc.police.uk/documents/S136%20Data%202015%2016.pdf).

[9] Home Office. *Policing and Crime Bill: Police Powers and Police and Crime Commissioners* Home Office, 2016 (https://www.gov.uk/government/publications/policing-and-crime-bill-police-powers-and-police-and-crime-commissioners).

[10] Murphy S, Irving C, Adam C, Waqar M. Crisis Intervention for People with Severe Mental Illness. Cochrane Library, 2015. 10.1002/14651858.CD001087.pub5 PMC7052814 26633650

[11] Paton F, Wright K, Ayre N, Dare C, Johnson S, Lloyd-Evans B, Improving outcomes for people in mental health crisis: a rapid synthesis of the evidence for available models of care. Health Technol Assess 2016; 20(3). 10.3310/hta20030 PMC4781004 26771169

[12] Kirst M, Pridham KF, Narrandes R, Matheson F, Young L, Niedra K, Examining implementation of mobile, police-mental health crisis intervention teams in a large urban center. J Men Health 2015; 24(6): 369–74. 10.3109/09638237.2015.1036970 26383041

[13] Bouveng O, Bengtsson FA, Carlborg A. First-year follow-up of the Psychiatric Emergency Response Team (PAM) in Stockholm County, Sweden: a descriptive study. Int J Men Health 2017; 46(2): 65–73.

[14] Lawn S, Smith A, Hunter K. Mental health peer support for hospital avoidance and early discharge: an Australian example of consumer driven and operated service. J Men Health 2008; 17(5): 498–508.

[15] Wyatt S, Aldridge S, Callaghan D, Dyke A, Moulin L. Exploring Mental Health Inpatient Capacity across Sustainability and Transformation Partnerships in England. NHS Midlands and Lancashire Commissioning Support Unit, 2019 (https://www.strategyunitwm.nhs.uk/sites/default/files/2019-11/Exploring%20Mental%20Health%20Inpatient%20Capacity%20accross%20Sustainability%20and%20Transformation%20Partnerships%20in%20England%20-%20191030_1.pdf).

