# Supporting people in mental health crisis in 21st-century Britain – ERRATUM

**DOI:** 10.1192/bjb.2020.143

**Published:** 2022-06

**Authors:** Andrew Molodynski, Stephen Puntis, Em Mcallister, Hannah Wheeler, Keith Cooper

In the original published article, country names were missing from the author affiliations. This has now been corrected.

## References

[ref1] Molodynski A, Puntis S, McAllister E, Wheeler H and Cooper K. Supporting people in mental health crisis in 21st-century Britain. BJPsych Bull 2020; 44(6): 231–32. Available from: 10.1192/bjb.2019.93. 31964448 PMC7684783

